# Vaccination in Switzerland

**Published:** 1877-04

**Authors:** 


Vaccination in Switzerland.—The *Echo* says. The strife concerning the utility of compulsory vaccination led some time ago to a general consultation of "all legitimate Swiss Physicians" upon this debated point. A committee was appointed, at the end of last year, to draw up a set of questions, issue them to all the members of the medical profession, and receive their answers. No fewer than 1,376 voting cards were sent out. Each card was divided into three columns headed, respectively "Yes," "No," and "Undecided." At the beginning of the month, 1,168 cards had been returned, each containing the specific suffrage of a qualified practitioner upon each of the questions, accompanied, in some cases, with a good deal of explanatory comment. The questions, which, after much debate, were reduced to five in number, were put in the following form: I. Does your experience incline you to the view that a thoroughly successful vaccination protects the subject against small pox, or at least against the severer forms of it, for a long series of years? To this question, 1,122 doctors replied "Yes," 22 "No," while 24 were "Undecided. II. Would you recommend the vaccination of healthy children? Answers —1,128 "Yes," 25 "No," 15 "Undecided." III. Would you recommend re-vaccination? Answers—1,083 "Yes," 60 "No," 25 "Undecided." IV. Do you consider that inoculation with retro-vaccinated cow-lymph offers such advantage that its use should be striven for? To this, 771 reply "Yes," the "Noes" rise to 213; 184 are "Undecided." V. Are you an advocate for the maintenance of the present compulsory vaccination? 1,010 replied "Yes," 133 "No," and 25 are "Undecided." Some of the answers are merely accompanied by pithy *marginalia*; others take the form of longer or shorter essays upon the whole question.—*British Medical Journal, March* 10, 1877.

